# Application of Mixed Reality to Ultrasound-guided Femoral Arterial Cannulation During Real-time Practice in Cardiac Interventions

**DOI:** 10.1007/s41666-023-00147-0

**Published:** 2023-09-08

**Authors:** Miguel Alonso-Felipe, Javier Manuel Aguiar-Pérez, María Ángeles Pérez-Juárez, Carlos Baladrón, Julio Peral-Oliveira, Ignacio J. Amat-Santos

**Affiliations:** 1 Data Engineering Research Group, School of Telecommunications Engineering, TSCIT Department, University of Valladolid, Valladolid, Spain; 2 Cardiology Department, Hospital Clínico Universitario de Valladolid, Valladolid, Spain; 3 Centro de Investigación Biomédica en Red de Enfermedades Cardiovasculares (CIBERCV), Instituto de Salud Carlos III, Madrid, Spain

**Keywords:** Femoral arterial, Cannulation, Puncture, Interventional cardiology, Mixed reality, HoloLens glasses

## Abstract

Mixed reality opens interesting possibilities as it allows physicians to interact with both, the real physical and the virtual computer-generated environment and objects, in a powerful way. A mixed reality system, based in the HoloLens 2 glasses, has been developed to assist cardiologists in a quite complex interventional procedure: the ultrasound-guided femoral arterial cannulations, during real-time practice in interventional cardiology. The system is divided into two modules, the transmitter module, responsible for sending medical images to HoloLens 2 glasses, and the receiver module, hosted in the HoloLens 2, which renders those medical images, allowing the practitioner to watch and manage them in a 3D environment. The system has been successfully used, between November 2021 and August 2022, in up to 9 interventions by 2 different practitioners, in a large public hospital in central Spain. The practitioners using the system confirmed it as easy to use, reliable, real-time, reachable, and cost-effective, allowing a reduction of operating times, a better control of typical errors associated to the interventional procedure, and opening the possibility to use the medical imagery produced in ubiquitous e-learning. These strengths and opportunities were only nuanced by the risk of potential medical complications emerging from system malfunction or operator errors when using the system (e.g., unexpected momentary lag). In summary, the proposed system can be taken as a realistic proof of concept of how mixed reality technologies can support practitioners when performing interventional and surgical procedures during real-time daily practice.

## Background and Significance

Virtual reality technologies (and its variations augmented reality and mixed reality) are playing an increasing prominent role in everyday life, from leisure to education and healthcare.

Virtual reality (VR) offers an immersive experience in an entirely artificial scenario. The new environment is entirely synthetic (computer-generated). The user must wear a VR headset which completely blocks perception of the surroundings. Virtual reality systems offer various degrees of immersive experiences, which can be classified in non-immersive, semi-immersive, and immersive [1].

Augmented reality (AR) offers an enhanced version of the real world by layering computer-generated virtual information (such as text, images, 3D models, music, video) over the real world [2] using a device combining a camera and a screen or a transparent headset. Mixed reality (MR) is an advanced form of the AR paradigm in which synthetic data is not only layered: instead, virtual objects are completely integrated within the real environment and can interact with it. The physical real world and the virtual computer-generated objects are difficult to distinguish [3].

Table 1 briefly describes virtual reality, augmented reality and mixed reality.

**Table 1:** Types of virtual reality technologies

Virtual reality
User interacts with an entirely computer-generated scenario Perception of real surrounding × Users need to always wear some type of device ✓ Example: Virtual traveling or virtual visits to museums. During the entire experience, the utilization of a wearable device, such as Meta Quest glasses, is required for scene rendering
Augmented reality
User interacts with an enhanced version of the real world containing computer-generated objects Perception of the real surrounding ✓ Users need to always wear some type of device × Example: Games in which virtual objects or creatures appear as if they are part of the player’s real environment. In such cases, individuals can actively participate in the game by donning augmented reality glasses like Magic Leap. Alternatively, they can integrate the game’s scenarios and objects into their environment without requiring additional wearable hardware. For instance, they can use the camera of a mobile phone to capture the real scene, overlay virtual objects on it and display the augmented view on the mobile phone screen
Mixed reality
User interacts with an enhanced version of the real world where physical and virtual objects are difficult to distinguish Perception of the real surrounding ✓ Users need to always wear some type of device × Example: Furniture fitting. Mixed reality allows users to interact with virtual objects, fostering a genuine sense of manipulation. In this particular case, advanced devices such as HoloLens 2 can be used to render furniture in their environment and place it according to their preferences. Another possibility is to use a mobile phone to view virtual objects on its screen while moving freely around the physical space

These technologies are opening new promising opportunities in the healthcare area as revealed by the literature [4–9].

More specifically, virtual reality–related technologies have started to be successfully used for the training of different techniques and interventions. Liang et al. [10] developed a simulation application for a mixed reality device that projected a human face displaying facial drooping (a symptom of stroke) onto a computerized training mannequin. Nursing students wearing a mixed reality device participated in the simulation to perform an assessment of their mannequin patient to identify the symptom of stroke and act accordingly. Students’ feedback was quite positive. Medellin-Castillo et al. [11] conducted three case studies to study haptics and virtual reality for the training of the orthognathic surgery (OGS), which is a very complex procedure that allows to correct a wide range of skeletal and dental irregularities, requiring surgical skills traditionally acquired through extended hands-on training using cadavers or models. Lin et al. [12] evaluated the use of virtual reality technologies to assist in the training of the lateral ventricle puncture necessary for external ventricular drain. Two groups of neurosurgical interns were trained with and without the system to validate its effectiveness. In general, these experiments provide initial evidence of the usefulness of virtual reality technologies in procedure training, showing an improvement of surgical skills, performance, and confidence of trainees, accompanied by a reduction of execution times and typical errors.

The integration of virtual reality technologies as real-time assistance in surgical procedures is more complex as a system malfunction can have undesirable catastrophic consequences and extremely accurate translation of virtual coordinates into the real world are required. Initial experiences are also found in the literature, although in a far more initial state than in training. Zhou et al. [13] presented a system to facilitate brachytherapy, a useful treatment for lung cancer, where the accuracy of needle positioning is critical. Using mixed reality for medical image fusion, the system allowed doctors to have an intuitive understanding of the patient’s tumor. The system was validated through a phantom experiment with good results for the needle insertion accuracy.

The use of mixed reality in surgeries and interventions is however very promising, as mixed reality allows physicians to easily interact with and manipulate physical and virtual environments and objects. An example is a pelvic ultrasound which is used for monitoring pregnancies, check for causes of abnormal bleeding or pain, or controlling intrauterine devices. When the doctor performs a pelvic ultrasound placing a transducer on the stomach or in the vagina, the medical images and related relevant information are presented on a screen. The physician must constantly turn his face to alternatively look at the patient where he is manipulating the instrument and the screen. The use of mixed reality in this procedure would allow to project ultrasound imaging data on the patient’s body.

Another good example are image-guided punctures performed during a variety of clinical procedures and surgeries, where accuracy is critical. Again, in these procedures, the practitioner needs to divide attention between the hand holding the puncture needle and a screen showing relevant medical images: another suitable scenario for the application of mixed reality. Table 2 summarizes different experiences where virtual reality technologies, and especially mixed reality, have been used to assist at punctures performed during clinical and surgical procedures in real-time practice in surgery or in phantom experiments.

**Table 2:** Examples of virtual reality technologies used to assist practitioners during punctures performed as part of clinical or surgical procedures

Reference	Type of virtual reality technologies	Objective
Lin et al. [12]	Virtual reality	Improving the lateral ventricle puncture skill which is an important step of external ventricular drain
Zhou et al. [13]	Mixed reality	Facilitate brachytherapy, which is an effective method for curing lung cancer, where the accuracy of needle positioning is vital and can influence the treatment effect
Uhl et al. [14]	Mixed reality	Facilitating the puncture of the common femoral artery in a phantom model
Li et al. [15]	Mixed reality, assisted by HoloLens glasses	Improving accuracy in respiratory liver tumor punctures in radiofrequency ablation (RFA)
Porpiglia et al. [16]	Mixed reality	Evaluating the feasibility of three-dimensional mixed reality holograms in establishing the access point and guiding the needle during percutaneous kidney puncture when performing percutaneous nephrolithotomy
Demerath et al. [17]	Augmented/mixed reality	Comparing the accuracy of augmented/mixed reality–guided versus frame-based stereotaxy-guided freehand drain placement in an intracerebral hemorrhage phantom model
Park et al. [18]	Mixed reality, assisted by HoloLens glasses	Improving procedural efficiency and reducing radiation dose for CT-guided lesion targeting in an abdominal phantom study
Davrieux et al. [19]	Mixed reality	Comparing ultrasound-guided needle punctures with the use of a mixed reality navigation system for percutaneous punctures
Jing et al. [20]	Virtual reality	Improving pediatric lumbar puncture clinical skill training of medical students through a virtual reality lumbar puncture model vs. the traditional rubber lumbar puncture model
Vrillon et al. [21]	Virtual reality	Improving the training of the practice of lumbar punctures through a three-dimensional video
Schneideret al [22]	Augmented reality, assisted by HoloLens glasses	Improving ventricular drain (a common neurosurgical procedure), providing guidance for ventriculostomy in a custom-made 3D-printed head model
Su et al. [23]	Virtual reality	Improving the implementation of lateral ventricle puncture operation through a haptic-based virtual reality simulator
Ulh et al. [24]	Mixed reality	Facilitating the puncture of the common femoral artery in a phantom model
Pratt et a.l [25]	Augmented reality, assisted by HoloLens glasses	Precise and efficient localization of perforating vessels during extremity reconstruction surgery using 3D vascular models
Tai et al. [26]	Virtual reality	Implementing a renal puncture surgical simulator to improve percutaneous nephrolithotomy surgery for renal calculi

## Objective

Virtual reality technologies open new and exciting possibilities in healthcare. Among virtual reality technologies, mixed reality outstands going one step beyond blurring boundaries between the real world and the virtual environment and allowing the user to interact with both the physical and the virtual objects, which become practically indistinguishable.

The objective of this work is to present a use case where mixed reality is used to assist physicians in quite complex interventional procedures, and specifically, in real-time ultrasound-guided femoral arterial cannulations. This use case should be taken as a good example of how virtual reality technologies can successfully support practitioners during clinical and surgical procedures.

Interventional cardiology is probably one of the most advanced fields in terms of minimally invasive approaches to complex cardiac diseases and the professionals are used to work with angiographic, echocardiographic, and computed tomography–based images simultaneously. Therefore, the additive value of mixed reality in this field is particularly relevant to increase situational awareness, integrate multimodal data, and provide hands-free system interfaces.

## Materials and Methods

### Method Overview

In this work, a mixed reality system, based in the HoloLens 2 glasses, has been developed to assist practitioners during real-time ultrasound-guided femoral arterial cannulations. In this surgical procedure, the doctor must look at a screen placed over the patient while holding an ultrasound scanner and a puncture needle. Although it is a common procedure, it involves a specific technique that requires challenging skills that must be mastered by practitioners in the field, and for this reason, this procedure may have difficulties for less experienced surgeons or operators. The main difficulty lies in the visualization of medical images and relevant related information and relative positioning of the needle used for the puncture in relation to the blood vessel. To fulfill these purposes, and to provide a helpful system, a mixed reality solution has been developed to assist the doctor during this surgical procedure, allowing to improve accuracy, and consequently reducing the time and cost of interventions, as well as the potential associated risks [27].

The physicians using the system participated in its design to produce a system adapted to their needs and requirements, taking into consideration working conditions at hospital, and the practitioners’ skills and attitude.

The main requirements posed by the operator and medical management staff can be summarized as follows:

- Cost-effective enough to be integrated in daily practice in surgery and procedures in public hospitals.
- Simple enough to be used by practitioners with extended experience in the procedure, i.e., femoral arterial cannulations, but with average technological skills.
- Reliable enough to be used in real-time surgeries and interventions, allowing surgeons and operators to increase accuracy when performing punctures in femoral arterial, as well as to reduce time consumed in the procedure.

Taking into account the above premises, a system divided into two modules was proposed, each of them with specific objectives: (1) transmitter module, which is responsible for sending medical images to HoloLens 2 and (2) receiver module, which is hosted in the HoloLens 2 and is responsible for rendering those medical images, placing them on a specific space location, and allowing the doctor to see and manage them in a 3D environment. These two main modules and their associated tasks are explained below.

#### Transmitter Module

This module is in the machine in charge of hosting the video transmission application. The main tasks of this module are the following:

1. Collecting a video stream of medical signals of interest.
2. Processing the video stream collected to make it suitable for the application.
3. Encapsulating and transmitting the video frames.

#### Receiver Module

This module, located in the mixed reality HoloLens 2 glasses, hosts the video reception application. Its tasks are divided into:

1. Decoding the flow of incoming frames.
2. Detecting and tracking an object of interest. In this case, a QR code is utilized as a straightforward and dependable target for tracking purposes across various objects, eliminating the necessity for training a model to track specific objects of interest. In this particular scenario, when employing an ultrasound scanner, the QR code will be affixed onto it for the tracking.
3. Projecting the video stream onto the object of interest.
4. Allowing the doctor to interact with the video projection (zoom in, zoom out, move it, close it).

### Materials: Hardware and Software Overview

Different hardware and software elements have been used for the transmitter and receiver modules:

#### Hardware

- Desktop computer for processing and programming 3D model virtualization.
  - Model: HP OMEN 875-1029NS.
  - CPU: Intel Core i9-9900K.
  - RAM: HyperX 64 GB DDR4-2666 SDRAM (4 x 16 GB).
  - GPU: NVIDIA GeForce RTX 2080Ti (11GB dedicated GDDR6).
- HoloLens 2 Mixed Reality device: As a visualization system for 3D modelling.
- Router 4G LTE Wi-Fi TP-Link TL-MR6400: As a common access point to the network for the communication between the computer and the HoloLens 2.
- Video capture card AVerMedia Live Gamer Mini 1080p 60fps: For the extraction and adaptation of images from different devices used in medical practice, that must be processed on the computer.
- Ultrasound scanner.

#### Software

- Visual Studio Code: IDE (Integrated Development Environment) for development and debugging code.
- Unity 2019.4.xf1: To visualize and configure the applications developed in the IDE.
- Unity packages: FMTEP Stream and AVPro Live. The first for the video transmission and reception service, and the second for projecting video onto a 3D object.
- Vuforia Engine: As Mixed Reality tool for object tracking (QR code in this application).

### System Explanation

**Fig. 1:**
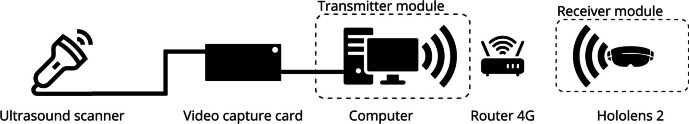
Hardware connection diagram. Note: Some connections have been removed from the diagram, such as power supply of video capture card, router 4G, and computer, to simplify it

For a better understanding, Fig. 1 illustrates how the different elements in the hardware system are connected.

First, the ultrasound scanner extracts images from the patient, which can be sent through HDMI (high-definition multimedia interface), if necessary, an adapter can be used for the output. In this research, a tablet with a commercial application has been used for image conditioning for the capture machine. Then, the video capture machine collects these images through the HDMI IN port and transmits them to the computer through the HDMI OUT port; the computer processes these images, encapsulates them in a UDP (User Datagram Protocol) datagram, and transmits them via broadcast on the network shared with the HoloLens 2. Finally, the HoloLens 2 receives the stream of UDP datagrams, decapsulates them, and renders them on a 3D object.

Once the hardware system has been explained, it is time to describe the two main modules that host the software applications: transmitter module and receiver module.

**Fig. 2:**
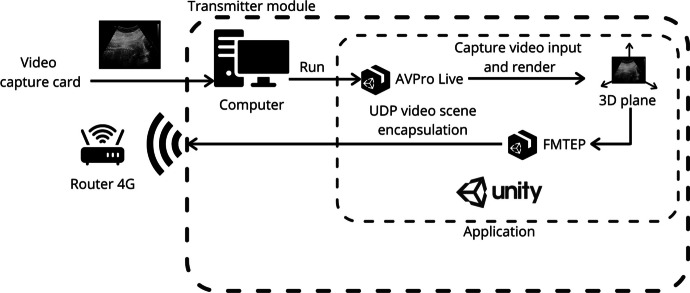
Functional diagram of transmitter module

The transmitter module is hosted in the computer. This module is responsible for the following tasks: receiving the flow of images (video stream) from the video capture device and the ultrasound scanner, processing those images to encapsulate them in UDP datagrams, and sending them broadcast to its local network so that they can be consumed by each web client connected to that network. To achieve this, the computer runs a Unity application which includes two main packages or assets called AVPro Live and FMTEP. AVPro Live allows the application to detect HDMI input video stream and render it onto a plane surface inside Unity 3D scene. Thus, FMTEP uses that plane surface to capture the video stream and encapsulate it in UDP datagrams to send it through the local network. To sum up, AVPro Live asset allows the application to detect and insert video from video capture card to FMTEP encapsulate and transmission system as shown in Fig. 2.

Once the computer sends the transmission, Router 4G must read the destination of the UDP datagrams, which is broadcast. That means the destination is each device connected to the network provided by Router 4G. That allows HoloLens 2 (receiver) to easily detect and extract the transmission without manual IP configuration, because every time a device leaves the local network, its IP is available for other devices (that also happens to HoloLens 2, which is the main destination of this system). For this reason, a configuration of the transmitter destination IP is needed every time the HoloLens 2 leaves the local network. So, to avoid manual configurations and to build an easy plug and play system, the destination will be broadcast (anyway, manual IP is available if the user wants to change the default configuration).

**Fig. 3:**
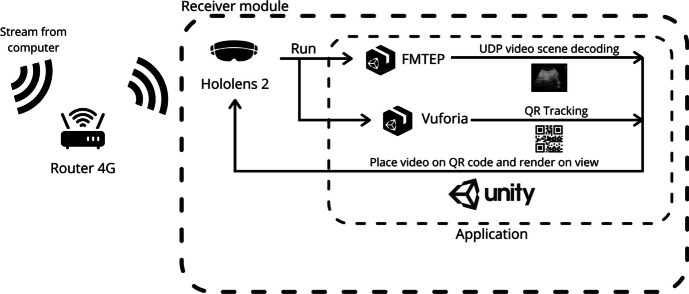
Functional diagram of receiver module

Finally, the receiver module has several functions to perform in parallel. On the one hand, it must render the content of the UDP datagrams in a plane, and on the other hand, it must detect a QR code and position the plane on it as shown in Fig. 3. The doctor can then place the QR code in an area of interest on which the image will be projected. An area of interest could be, for example, the ultrasound scanner. Thus, the ultrasound images could be presented right above the patient and follow the ultrasound scanner using QR tracking. Furthermore, the doctors can lock the position of the plane (thereby terminating the tracking) by utilizing a button situated at the top of the window. This enables them to conveniently reposition the plane to a more suitable location when needed, simply by grabbing and moving it manually. Additionally, they can reactivate the tracking functionality by pressing the same button.

Another consideration is that, although real-time streaming is necessary for this medical practice, it is important to find a balance between resolution and delay depending on the procedure’s significance. In this context, real-time capability is of great relevance, as any latency, such as screen freezing or delays, could potentially hinder the doctor’s ability to effectively perform the puncture and compromise patient safety.

To achieve this goal, UDP is selected. UDP is a lightweight and fast protocol, and base64 encoding is used to simplify image data transfer. However, higher resolution requires more data to be transmitted, which compromises the stability of the connection for applications where resolution is crucial. To meet this challenge and achieve a near real-time streaming experience, it is crucial to keep the latency near to 200 ms for any given image resolution. In this case, for a 640 × 480 image resolution (which turned out to be sufficient for the medical practice of arterial puncture), an average latency of 200 ms is achieved, with occasional outliers reaching around 250 ms, primarily due to wireless connectivity issues.

It is crucial to incorporate efficient logic for encoding, transmitting, decoding, and rendering data in order to minimize latency, considering the potential inconsistency of connection integrity. To achieve a more efficient system, it is highly recommended to leverage the computational power of a computer or laptop instead of burdening the HoloLens 2 with processing requirements.

### Validation: Pilot Study

The developed mixed reality system, based on HoloLens 2 glasses, was validated as a proof of concept in a pilot study conducted in a tertiary public hospital in central Spain. A single use case was selected to focus validation on a single task: the system was employed to facilitate ultrasound-guided femoral arterial cannulation in interventional cardiology procedures. In the period between November 2021 and August 2022, 9 patients with severe aortic stenosis selected for trascatheter aortic valve replacement (TAVR), with planned femoral access in the pre-intervention CT-Scan, were included in the study. Two different practitioners carried out the interventions (6 and 3 interventions each).

This pilot study was reviewed and approved by the local Ethics Committee (*Comité de Ética de la Investigación con Medicamentos del Área de Salud Valladolid Este*) with code PI-21–2183. Ethical and regulatory concerns have been carefully considered: although the system is only a visualization component (it does not process any medical information, it only presents a video stream in a more convenient way than a conventional 2D screen), there are critical aspects in the interaction with the system (such as lag or image aspect ratio) that could theoretically influence the medical practice in specific applications. For this reason, the system cannot currently be employed outside a regulated research project.

## Results

**Fig. 4:**
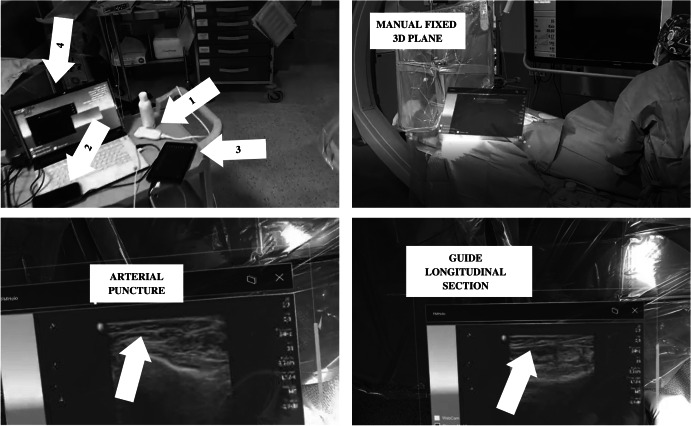
Different views of the system. Top left quadrant shows the main hardware components of the transmitter module: (1) ultrasound scanner, (2) video capture card, (3) tablet, and (4) laptop. Top right quadrant shows a plane rendering ultrasound scanner images fixed manually by the doctor over the patient using the button placed on the top right corner of the window (next to the cross button). Bottom-left quadrant shows an arterial puncture assisted by the system. Bottom-right quadrant shows the guide introduced into the artery from a longitudinal section view

Figure 4 shows a picture of the transmitter module setup and different moments of the use of the system in real-time ultrasound-guided femoral arterial cannulations in interventional cardiology procedures. This picture shows how helpful the system can be in a real-life environment.

As shown in Fig. 4 top-right quadrant, the doctor may want to manually handle the plane. In this scene, the doctor fixes the plane over the patient. According to the requirements of this system established by the practitioners, some situations need reallocating the plane on different places. This feature may be helpful to clean the view during an intervention or to allocate the plane in a more comfortable place for the doctor depending on different factors such as the scenario illumination and textures of the background.

Furthermore, Fig. 4 bottom-right and -left quadrants show two different situations in which the interventional cardiologist follows the puncture and the guide used to proceed in the safest possible manner while using the system. As shown, the cardiologist can operate looking at the point where the puncture is performed, and at the ultrasound scan, simultaneously, reducing the risks and time of the intervention. Thus, according to the practitioners’ report, the possibility of operating looking at the point of interest is more intuitive and comfortable than the old method, which was based on a screen located vertically above the patient, that forced the doctor to look away from the operating table and the patient, as shown in the top-right quadrant of Fig. 4.

As a proof of concept, the main objective of the study was to demonstrate the feasibility of the approach. Nine out of 9 punctures were considered successful: no need to re-access, no recorded adverse events associated with puncture (bleeding). After the experience, the two participating interventional cardiologists rated the experience according to 5 subjective parameters, presented in Table 3.

**Table 3:** Average subjective score given by interventional cardiologists to the mixed reality system applied to ultrasound-guided puncture. 1 is the lowest possible score, 5 is the highest possible score

Parameter	Average score
Ease of use	4.5
Responsiveness	4.5
Image quality	4
Lack of technological burden	4.5
Headset comfort	3.5

In the post-experience interview, operators mentioned two main limitations:

- Low image contrast in a brightly illuminated environment.
- Headset could be uncomfortable to wear for long periods of time (such as entire shifts in the cath lab).

## Discussion

The practitioners using the mixed reality system, based in HoloLens 2 glasses, also participated in a focus group-based SWOT (strengths, weaknesses, opportunities, and threats) qualitative analysis. In this analysis, related medical management staff were also involved. Main conclusions are summarized in Table 4.

**Table 4:** SWOT analysis of the mixed reality developed system

Strengths
Easy to use Reliable Real-time Reachable Cost-effective
Opportunities
Reduction of operating time Better control of typical errors associated to the procedure Medical information and images produced can be broadcasted and practitioners can be assisted by more expert colleagues Medical information and images produced can be broadcasted and used for training purposes with interns or novice practitioners integrated in a ubiquitous e-learning system
Weaknesses
Digital divide for practitioners used to more classical surgical procedures and techniques not involving the use of virtual reality technologies
Threats
Some practitioners are afraid of potential system errors or of the learning curve of this new approach

The thoughts of the users revealed by the SWOT analysis confirmed their perception of that their requirements have been met.

As main strengths, practitioners highlighted the possibility to be used during real-time practice in interventions, as well as its reliability, as the system allowed them to perform punctures in femoral arterial cannulations with high accuracy. Moreover, practitioners confirmed that the system has been easy to use and helpful. Medical management staff added that the system was reachable for the public health system as it can be deployed and managed at a reasonable cost.

Regarding the opportunities, practitioners believe that its use in interventional cardiology can reduce operating times and allow a better control of the procedure, especially of the typical mistakes made by interns and novice doctors. Operators involved in the training of medical students also highlighted that the system produce real-time valuable medical imagery that could be used for training purposes in the context of a ubiquitous e-learning system, even with students abroad. The possibility to broadcast real-time medical imagery would also open the possibility to ask for advice of more expert colleagues, even from other hospitals, during interventions, which can be especially useful for less experienced practitioners, or in the case of complex cases.

Regarding the weaknesses and considering the possibility of using the system in other hospitals, the practitioners suggested that interventional cardiologists used to more classical procedures, not involving the use of virtual reality technologies, may have difficulties adopting the new system. They highlighted that there was a digital divide affecting mainly to older practitioners, and remarked that, in their case, they have both volunteered to use the system. Related to this fear, practitioners suggested as a threat, which some colleagues may be afraid of potential system errors or of the learning curve of this new approach.

## Conclusion

In conclusion, the mixed reality system developed, based in HoloLens 2 glasses, is reliable and simple enough to be used by practitioners during real-time practice in interventional cardiology. This was confirmed by practitioners’ feedback during a qualitative analysis of the system. The system has been used to assist physicians in femoral arterial cannulations in up to 9 interventions in a large public hospital in central Spain since November 2021. The system is also cost-effective enough to be incorporated into the public health system. As a summary, the proposed system is a realistic proof of concept of how virtual reality technologies, and more specifically mixed reality, can successfully support practitioners during clinical and surgical procedures.

QR code has proven to be an effective method for tracking various objects in well illuminated scenarios like surgery rooms. One potential direction for future investigation would be to explore object tracking utilizing other HoloLens 2 sensors, such as LiDAR (light detection and ranging), and the potential opportunities it presents for low light conditions. In addition, the main line of research open in order to transfer the prototype into clinical practice is to conduct a randomized clinical trial with a twofold purpose:

• First, to quantitatively characterize the potential clinical advantages for the patient and the practitioner (shorter intervention times and radiation).

• To obtain solid scientific evidence on which to base future potential certification claims to regulatory authorities.

## Data Availability

There are no new data associated with this article.
